# Supplementary material for: Causal interplay between lactose intolerance and gut microbiota: a combined bidirectional Mendelian randomization and in vivo validation study
Source: Front Nutr. 2026 Jun 1;13:1803337. doi: 10.3389/fnut.2026.1803337 (PMC13265576; doi:10.3389/fnut.2026.1803337)
Supplement: Supplementary file 1 [file Data_Sheet_1.zip › supplementary materials/Forward/leave one out cross validation/ebi-a-GCST90027504.finngen_R12_E4_LACTONAS.pdf]

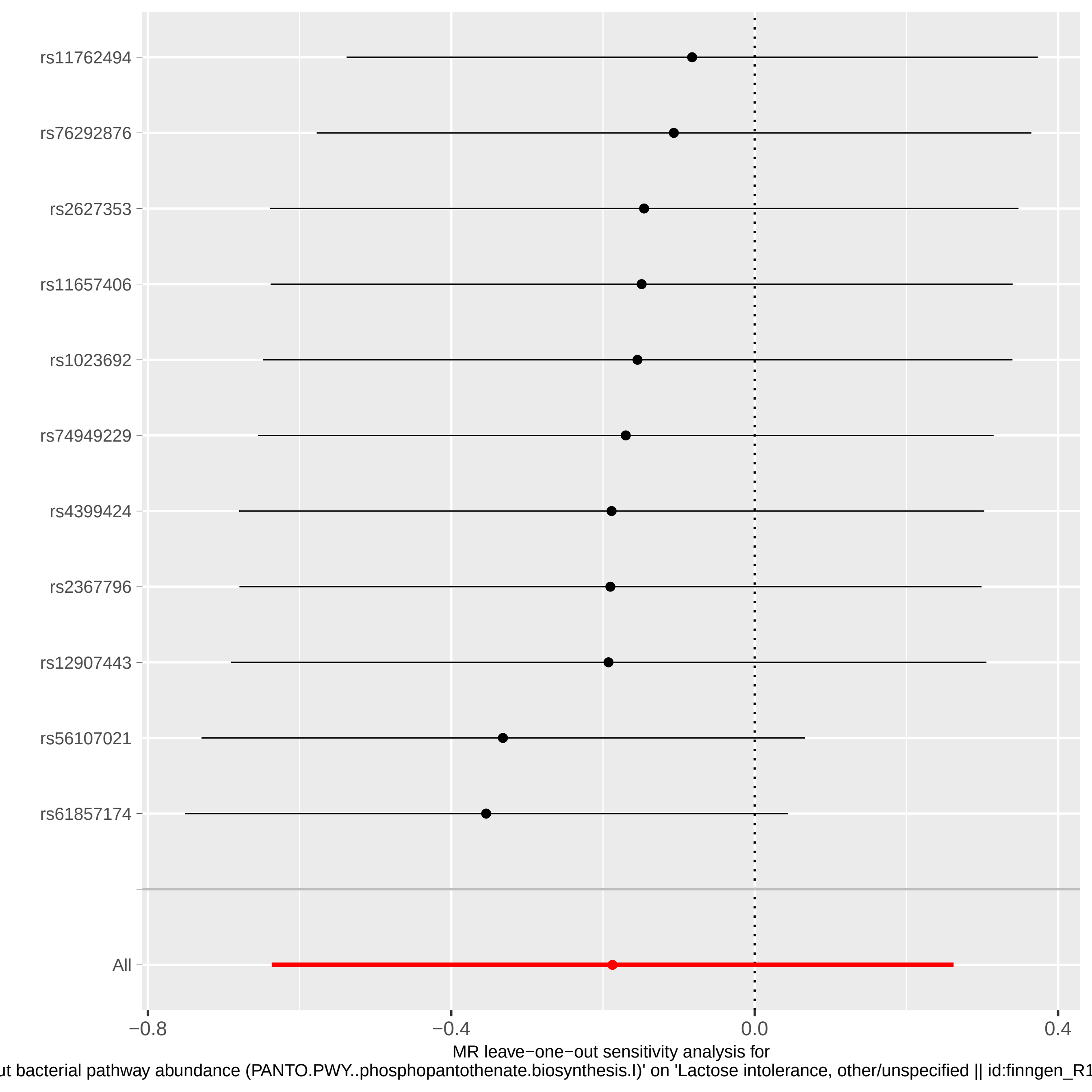

rs11762494
rs76292876
rs2627353
rs11657406
rs1023692
rs74949229
rs4399424
rs2367796
rs12907443
rs56107021
rs61857174
All
−0.8
−0.4
0.0
0.4
MR leave−one−out sensitivity analysis for
ut bacterial pathway abundance (PANTO.PWY..phosphopantothenate.biosynthesis.I)' on 'Lactose intolerance, other/unspecified || id:finngen_R1
